# Supplementary figures and images for: Nonlinear modeling of oral glucose tolerance test response to evaluate associations with aging outcomes
Source: PLoS One. 2024 May 16;19(5):e0302381. doi: 10.1371/journal.pone.0302381 (PMC11098391; doi:10.1371/journal.pone.0302381)

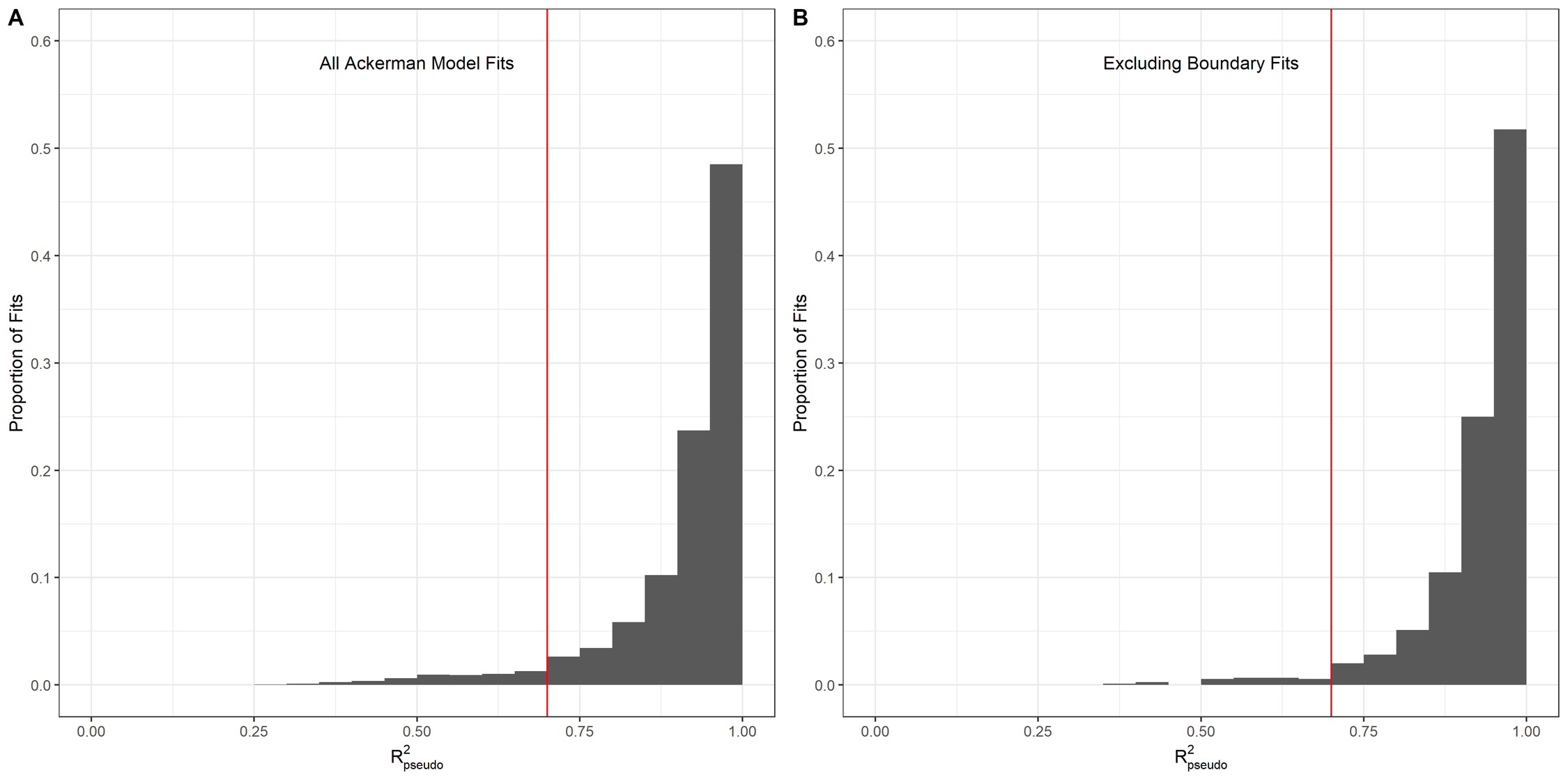

Supplement: S1 Fig — Red vertical line indicates Rpseudo2=0.7. Panel A—Ackerman model fits from all OGTT curves. Panel B—Ackerman model fits excluding boundary fits. (TIF) [file pone.0302381.s001.tif]

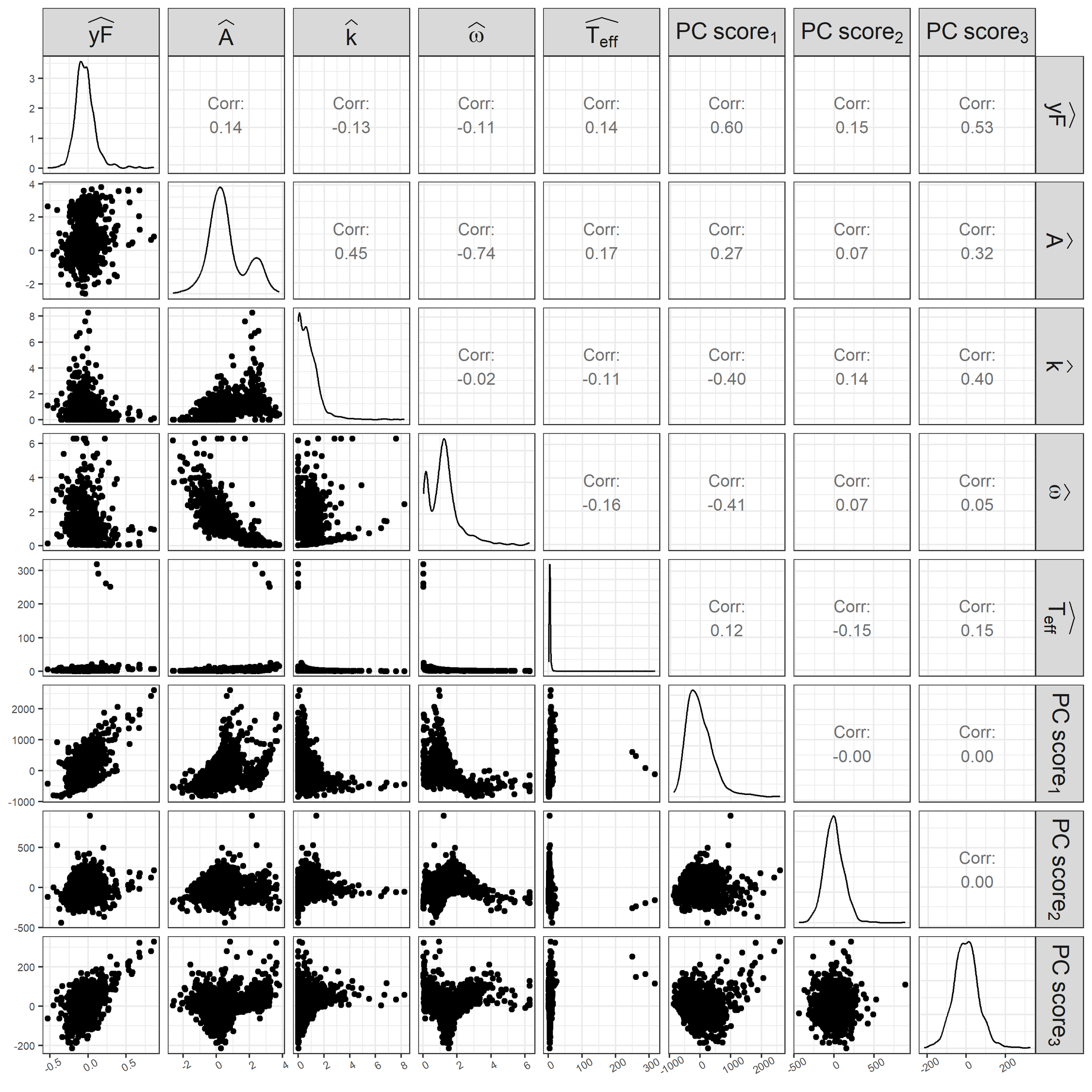

Supplement: S2 Fig — (TIF) [file pone.0302381.s002.tif]

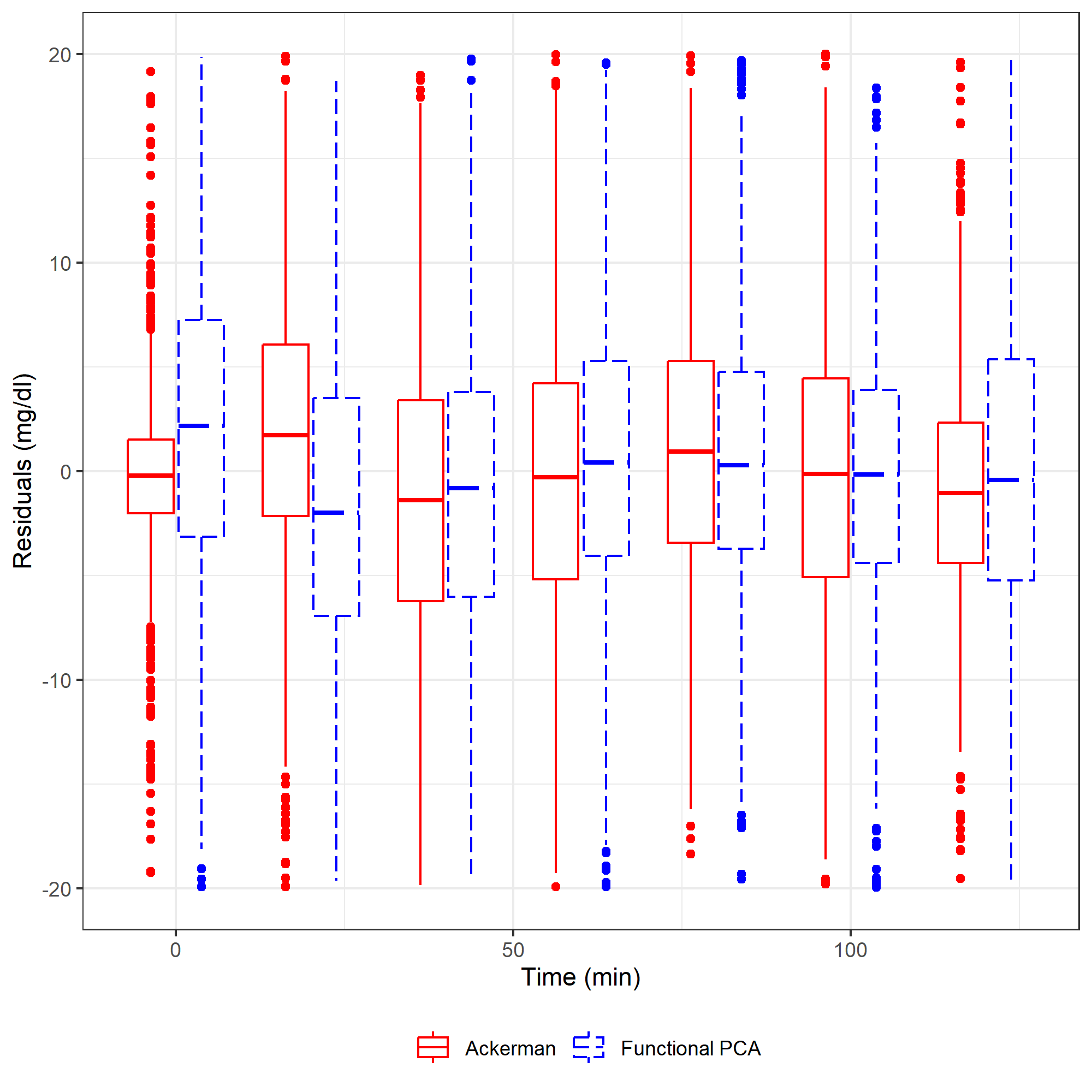

Supplement: S3 Fig — Outliers above 20 mg/dl and below −20 mg/dl were excluded from the plot but were included in constructing the boxes. (TIF) [file pone.0302381.s003.tif]
