# Supplementary material for: Nonlinear modeling of oral glucose tolerance test response to evaluate associations with aging outcomes
Source: PLoS One. 2024 May 16;19(5):e0302381. doi: 10.1371/journal.pone.0302381 (PMC11098391; doi:10.1371/journal.pone.0302381)
Supplement: Attachment — Submitted filename: comments.docx [file pone.0302381.s004.docx]

Frailty: define the term –line23

Line49- needs reference

Line57- what are the 4 parameters

Line 63 -vardhan et al which year?

Line65-66- reference-“We found greater variability in the shapes of OGTT curves between 65 individuals than expected”.

Line80-Specify “these”.- Does it mean the curves?
