# Supplementary material for: Nonlinear modeling of oral glucose tolerance test response to evaluate associations with aging outcomes
Source: PLoS One. 2024 May 16;19(5):e0302381. doi: 10.1371/journal.pone.0302381 (PMC11098391; doi:10.1371/journal.pone.0302381)
Supplement: Attachment — Submitted filename: Response to Reviewers.docx [file pone.0302381.s005.docx]

Dear Editor,

We hope this email finds you well. The authors would like to express our gratitude the editor, Burak Bayraktar, and the two reviewers for their insightful feedback, corrections, and questions. The manuscript has been greatly improved by your contributions. We have attached our responses following this letter and in the revised and marked up manuscripts.

We appreciate your consideration of this revised manuscript for publication in PLOS ONE.

| Point being addressed | Response | Changes Made | Additional notes |
| --- | --- | --- | --- |
| EDITOR: 1. Please ensure that your manuscript meets PLOS ONE's style requirements, including those for file naming. The PLOS ONE style templates can be found at  https://journals.plos.org/plosone/s/file?id=wjVg/PLOSOne_formatting_sample_main_body.pdf and  https://journals.plos.org/plosone/s/file?id=ba62/PLOSOne_formatting_sample_title_authors_affiliations.pdf |  | 1. format changed to double spacing  2. Fig 5 title altered to include a period at end  3. Removed spaces from figure files’ names | If there are any additional formatting requirements we missed, we am happy to update the manuscript again |
| EDITOR: 2. Note from Emily Chenette, Editor in Chief of PLOS ONE, and Iain Hrynaszkiewicz, Director of Open Research Solutions at PLOS: Did you know that depositing data in a repository is associated with up to a 25% citation advantage (https://doi.org/10.1371/journal.pone.0230416)? If you’ve not already done so, consider depositing your raw data in a repository to ensure your work is read, appreciated and cited by the largest possible audience. You’ll also earn an Accessible Data icon on your published paper if you deposit your data in any participating repository (https://plos.org/open-science/open-data/#accessible-data). | We cannot do this due to restrictions with the BLSA’s IRB agreement | none |  |
| 3. Please include your full ethics statement in the ‘Methods’ section of your manuscript file. In your statement, please include the full name of the IRB or ethics committee who approved or waived your study, as well as whether or not you obtained informed written or verbal consent. If consent was waived for your study, please include this information in your statement as well. |  | 1. Removed text “Participants provide written informed consent at each visit. The BLSA protocol has been approved by the Intramural Research Program of the US National Institute on Aging and the Institutional Review Board of the National Institute of Environmental Health Sciences. All data used in this study were fully anonymized by the National Institute on Aging before being provided to the study team.” (lines 92-97)  2. Added text “Data used in this manuscript are from the BLSA which is approved by the Institutional Review Board of the National Institute of Environmental Health Sciences under protocol #03-AG-0325. BLSA participants given written informed consent at each study visit. The National Institute of Aging provided the data used in this manuscript to the study team upon review of the study plan and completion of a data use agreement. The data provided by the BLSA to the study team were retrospective and anonymized and thus were acknowledged to not be human subjects research by the study team's IRB---the Johns Hopkins Medicine IRB.” (lines 105 to 115) |  |
| EDITOR: 4. We note that you have indicated that there are restrictions to data sharing for this study. PLOS only allows data to be available upon request if there are legal or ethical restrictions on sharing data publicly. For more information on unacceptable data access restrictions, please see http://journals.plos.org/plosone/s/data-availability#loc-unacceptable-data-access-restrictions.  Before we proceed with your manuscript, please address the following prompts:  a) If there are ethical or legal restrictions on sharing a de-identified data set, please explain them in detail (e.g., data contain potentially identifying or sensitive patient information, data are owned by a third-party organization, etc.) and who has imposed them (e.g., a Research Ethics Committee or Institutional Review Board, etc.). Please also provide contact information for a data access committee, ethics committee, or other institutional body to which data requests may be sent.  b) If there are no restrictions, please upload the minimal anonymized data set necessary to replicate your study findings to a stable, public repository and provide us with the relevant URLs, DOIs, or accession numbers. For a list of recommended repositories, please see  https://journals.plos.org/plosone/s/recommended-repositories. You also have the option of uploading the data as Supporting Information files, but we would recommend depositing data directly to a data repository if possible.  We will update your Data Availability statement on your behalf to reflect the information you provide. | We cannot upload our data due to the consent form used in the BLSA. We have modified our Data Availability statement to reflect this | New Data Availability statement: This paper relies on data from the Baltimore Longitudinal Study of Aging (BLSA), which are not publicly available due to the consent form language that the BLSA participants agreed to. The National Institutes of Health IRB supervises the BLSA and must approve any data release. To request data from the BLSA, please visit the BLSA website [https://www.blsa.nih.gov]/ and fill out a form. The BLSA Data Sharing Proposal Review Committee, which evaluates and approves all data requests/releases, meets once a month. |  |
| EDITOR: “The BLSA continuously enrolls healthy adults aged 20 years and older. Participants undergo extensive, 3-day testing every 1-4 years, with older participants visiting more frequently. Participants provide written informed consent at each visit.” What is the content of BLSA? For readers who don't know, tell us about it in a few sentences. | Thanks for the suggestion. We have added some additional context. | 1. Removed “The BLSA continuously enrolls healthy adults aged 20 years and older.” (lines 90-92)  2. Added “The BLSA is a community-based cohort study that continuously enrolls healthy volunteers aged 20 years and older who live within two hours driving from Baltimore, Maryland \cite{Ferrucci2008}. The BLSA follows participants for life. The study was designed to answer mechanistic questions about aging and the transition from health to disease with age.” (lines 98 to 102 ) |  |
| REVIEWER 2: Frailty: define the term –line23 | Added | added: “---those demonstrating decreased resilience to stressors, as defined by the Fried frailty index---” (lines 23 to 24 ) |  |
| REVIEWER 2: Line49- needs reference | Added the references from the subsequent two sentences | Added citations for Bolie1961, Brubaker2007, Man2002, Gatewood1968, Ackerman1964 (line 51 ) |  |
| REVIEWER 2: Line57- what are the 4 parameters | added additional text | Added “---one which estimates fasting glucose, another which controls the rate of an exponential decay function, one which controls the sinusoidal pattern of the glucose curve, and the last influences the amplitudes of the glucose curve oscillations.” (lines 60 to 63 ) |  |
| REVIEWER 2: Line 63 -Varadhan et al which year? | Modified two sentences to include the year | Removed “We set out to apply Varadhan et al.'s framework” (lines 37 to 38)  Added “We applied the framework laid out in Varadhan et al. 2008” (lines 38 to 39 )  and  Changed “In applying Varadhan et al.'s framework,” (line 63)  to  “In applying the framework described in Varadhan et al. 2008,” (line 68) |  |
| REVIEWER 2: Line65-66- reference-“We found greater variability in the shapes of OGTT curves between 65 individuals than expected”. curves between 65 individuals than expected”. | Modified the text to clarify that we were not comparing the amount of variability that we observed to a known standard | Removed “We found greater variability in the shapes of OGTT curves between individuals than expected.” (lines 65-66)  Added “We found significant variability in the shapes of OGTT curves between individuals.” (lines 70 to 71) |  |
| REVIEWER 2: Line80-Specify “these”.- Does it mean the curves? | Modified the sentence to make it more clear | Removed “and reports metrics by which to identify these.” (lines 79-80)  Added “. We termed the Ackerman model fits associated with these curves ``inadequate fits'' and we present the criteria we devised to identify these fits in the results section.”  (lines _86 to 88 _) |  |
| REVIEWER 1: For the description of the Ackerman model, what do the parameters in the model (l1-l6) represent. I could also not relate the formulation of the Ackerman model presented to in this to the equations presented in the references 1965 paper, namely how l5 and I related to the K term presented in that paper. | Added descriptions for l1-l6. In the 1965 paper, they show the equations for the change in glucose and insulin compared to baseline levels. Since l2 and l5 represent average rates of release independent of glucose/insulin (respectively), they drop out. The K term in the 1965 paper is the rate or injection of H per unit blood volume. Since we using OGTT data, there is no insulin injected---that is why we do not have a K term. In other GTT studies, such as IVGTT, K could be some non-zero function. | Added: “$l_1H$ is the average rate of insulin removal independent of glucose, $l_2$ is the average rate of release of insulin from the pancreas independent of glucose, $l_3Y$ is the net increase in the rate of release of insulin due to glucose, $l_4Y$ is the average rate of glucose removal independent of insulin, $l_5$ is the average rate of release of glucose into the blood, and $l_6H$ is the net increase in the average rate of glucose removal from the blood due to insulin \cite{Ackerman1964}” (lines 166 to 171) |  |
| REVIEWER 1: Secondly, are equations 1 and 2 printed correctly? I do not understand why I, which is reported to describe the rate of appearance of glucose from the gut, negatively contributed to the rate of change of plasma glucose. | Thank you for pointing out the mistake in equation 2. It has been corrected. Equation 1 was correct (pg 205 in Ackerman 1964) | changed “-I” to “+I” in equation 2 |  |
| REVIEWER 1: The authors report that the model is fully identifiable. Firstly, what do the authors mean by identifiable? Do they refer to structural identifiability which is a feature of the model structure itself or practical identifiability which characterised the ability to estimate unique parameters given the available data? How was this model identifiability determined? The authors later report that several models are classed as inadequate due to “near redundant” fits, which to me sounds like an identifiability issue. Was practical identifiability assessed for each individual or for the population average? | Thank you for these excellent points. For that sentence (line 196), we were referring to structural identifiability. We have modified this line to reflect this. We did find practical identifiability to be an issue, and we were referring to this with the section discussing “near redundant” fits. We have added text to this section to clarify. Practical identifiability was assessed for each individual using profile likelihoods. We found that for the majority of our OGTT curves, the Ackerman model was practically identifiable. For a subset of the curves, we found that multiple sets of parameters produced equal (or near-equal) fits of the data in terms of likelihoods. The profile likelihoods for this subset revealed nearly flat profile likelihoods near the optimum when profiling over A_i, k_i, and w_i. However, we found this subset of curves was identified as being problematic from our extrapolated fit and boundary fit criteria. Therefore, we are essentially throwing out the parameters estimated from these practically non-identifiable fits before running the stage-two regression models since they are unreliable. We have added some text to describe that we examined profile likelihoods to evaluate practical identifiability. | removed “fully” line 218 added “structurally” (line 219 )  added: “These fits are caused by practical non-identifiability.” (lines 351-352)  added: “In a sensitivity analysis, we created profile likelihood plots for each OGTT to examine for practical non-identifiability of the Ackerman model. We found several instances of practical non-identifiability, as evidenced by flat profile likelihoods near profile maxima. However, these OGTT curves were found to be a subset of the inadequate fits we already identified, so these Ackerman model fits were already excluded from the stage-two models.”  (lines 363 to 368) |  |
| REVIEWER 1: How many models are lost/deemed inadequate due to each of the three criteria outlined in the results section, namely parameters hitting boundaries, extrapolated fits, and pseudo-R-squared? How does this compare to the fPCA approach, are any individuals lost in this approach? | The number of inadequate fits for each type were/are listed in lines (394 to 396 first submission, 435 to 438 revised submission). This paragraph was not clearly denoted as separate from the section on low-R2_pseudo fits previously, and this has been revised.  Using fPCA, we did not create a separate classification for predictions which poorly fit the observed data as we did with the Ackerman model. fPCA is more flexible than the Ackerman model, so we did observe any curves as obviously not biologically plausible as we did with the Ackerman model. Furthermore, the fPCs do not have the same clear interpretation as the Ackerman model parameters do (e.g. k_i<0 would indicate increasing oscillations in blood glucose). However, the predicted OGTT curves from fPCA did not always match the data closely. We added text to address your latter question as well as to clarify the meaning of explaining 99% of variability in the smoothed OGTT curves. | added “the smoothed” (line 442-443 )  added “smoothed” (line 646)  added “This indicates that the variability between the predicted OGTT curves using fPCA and the observed OGTT curves is primarily due to the data smoothing process---not due to dropping higher order eigenfunctions.” (lines 646 to 649 )  added: “Unlike with the Ackerman model, we did not intentionally identify poor-fitting predicted OGTT curves fit using fPCA. First, the eigenfunctions do not have a direct biological interpretation like the Ackerman model parameters do. While certain combinations of PC scores could lead to predicted OGTT curves which are not biologically plausible, this cannot be determined by examining only one PC score in isolation. Second, each PC score is influenced by all of the other curves in the data set. The Ackerman model is fit independently to each individual's OGTT curve so isolating inadequate fits does not impact other fits.” (lines 467 to 474) |  |
| REVIEWER 1: My understanding is that Y_Fi corresponds to the basal glucose level in the plasms, I also understand that this parameter is being estimated from data in this study. Would it not be possible to directly infer a value for this parameter based on the fasting glucose value or could potentially using some form of regularization aid with the identification of an identifiable parameter set for all individuals? Do the authors have specific reasons to not use the measured fasting glucose concentration as a proxy for Y_Fi? | It would be possible to use the glucose measurement at time 0 directly for Y_Fi rather than estimating it. Then, rather than estimating 4 parameters using 7 data points, we would estimate 3 parameters from 6 data points. By instead estimating Y_Fi, we are in essence doing a regularization by allowing the time points at 20, 40,…,120 minutes influence the value for Y_Fi. In turn, we are able to use the time point at 0 minutes in the estimation of A_i, k_i, and w_i. We experienced the most difficulty with estimating A_i and w_i: A_i and w_i could be varied inversely to provide nearly identical SSRs with significant changes in A_i and w_i. We also had trouble with fitting k_i for some OGTT curves. For some fits, the k_i-hat was estimated to be 0 even though biologically this is not meaningful. Because of these difficulties, and the relative ease of fitting Y_Fi, we estimated Y_Fi rather than using the glucose measurement at time 0. While this does mean there is nearly always some model error at time 0, the tradeoff for estimating A_i, k_i, and w_i was worth it in our opinions. | No changes made |  |
| REVIEWER 1: The authors report using the nls function in R, from their description I assume this is a local least squares solver where parameter fitting was run until a ‘satisfactory’ fit was obtained. What is the distribution of the number of initialization required before an acceptable model fit is found? Did the authors to any testing to evaluate if a global optimum had been obtain? Moreover, related to parameter identifiability, was there any test performed to ensure the model had converge to a unique local minimum? Namly, were any checks in place to demonstrate that the best fitting model had been obtained, or just that an acceptable model fit have been achieved. What might the implication be for the interpretability of the parameter estimates? | Yes, nls is a local least squares solver. We found that it typically only required one initialization to find a satisfactory fit, or else a satisfactory fit was not found. About 700 fits were stopped after the first initialization, around 400 after the 1000^th^ iteration (the maximum number), and 8 occurred in between (4 at the 2^nd^, 1 at the 7^th^, 1 at the 13^th^, 1 at the 38^th^, and 1 at the 59^th^). All of the fits that were obtained at the maximum iteration were identified as inadequate fits except for 19. Examination of these 19 fits showed large glucose values after 20 minutes, which resulted in large residual sums of squares---one of the stopping criteria we used.  We did not test that the fits reached a global optimum. We did however examine profile likelihoods as described in our previous response. We also did a sensitivity analysis using nls.multstart, an automated multiple-start local solver. We did not find this produced different optimal fits for the adequate fits. For many of the inadequate fits, each run of nls.multstart produced a different MLE. This led us to conclude that the inadequate fits, particularly the boundary fits, could not be meaningfully interpreted. | No changes made | If you believe it is important that we add text regarding this, we can |
| REVIEWER 1: With regard to the extrapolated fits, the authors report that model fits where absolute difference between the maximum predicted glucose concentration and observed glucose concentration exceed a specified threshold. However, if I understand correctly the OGTT procedure makes use of discrete sampling (0,20,40,60,80,100,and 120 minutes) is it not possible that the true glucose peak occurs between two sampling points and consequently many be higher than what has been observed? May this exclusion threshold be too stringent? | This is an excellent point. It is probably the case for many of the OGTT curves that the true peak occurred sometime between the sampling points. We selected the exclusion threshold of over 10% relative error based on Fig 3 (10%, indicated by the grey lines, seems to contain most of the fits) and visual inspection of the individual fits falling around this 10% relative difference mark.  In practice, we found it much more common that the Ackerman model underpredicted the maximum observed glucose than overpredicted it---there were 9 cases the maximum predicted was deemed too large, and 22 cases it was deemed too small. Those 22 fits would be even worse at estimating the true peak glucose compared to the maximum observed glucose for the reason you pointed out. For the 9 fits, it is true the difference between the predicted and true peaks could be less than 10% relative difference even though the difference between the predicted and observed peaks was greater than 10%. However, in most of these 9 cases, the relative difference was quite a bit more than 10% (indicated by the distance from the upper grey line in Fig 3). The 10% mark may have been too stringent for a few of these 9 fits, but visual inspection of the two fits closest to this 10% mark showed significant overprediction compared to the observed data. Although for our specific data we do not think the gap between observed and true peaks was an issue, it is an excellent point and should be mentioned in case any researchers would like to apply a similar methodology in a new context or with different data. We have added a note of the potential issue. | added: It should be noted that an individual's peak glucose during an OGTT likely occurs in the time between two of the observed measurements. Thus, the observed peak glucose is almost certainly lower than the true peak glucose. Care should be taken to ensure that a screening criterion such as that presented in Eq~\ref{eq:extrapolated fit defn} is not too stringent given that the true peak glucose is not measured. For this study, we did not find this to be an issue as the Ackerman model typically underpredicted the peak glucose when the predicted and observed peaks varied by more than 10\% relative difference.  (lines 404 to 410) |  |
